# Sicca Symptoms in Parkinson's Disease: Association with Other Nonmotor Symptoms and Health-Related Quality of Life

**DOI:** 10.1155/2020/2958635

**Published:** 2020-02-12

**Authors:** Tino Prell, Denise Schaller, Caroline Perner, Otto W. Witte, Julian Grosskreutz

**Affiliations:** ^1^Department of Neurology, Jena University Hospital, Jena, Germany; ^2^Center for Healthy Aging, Jena University Hospital, Jena, Germany; ^3^Center for Immunology and Inflammatory Diseases, Massachusetts General Hospital, Harvard Medical School, Charlestown, MA, USA

## Abstract

**Background:**

Frequently used nonmotor scales do not cover all aspects of dysautonomia in Parkinson's disease (PD). This study explores the association between autonomic symptoms and sicca symptoms with other nonmotor symptoms and health-related quality of life (QoL) in PD.

**Methods:**

Autonomic symptoms (Survey of Autonomic Symptoms, SASs), motor function (Movement Disorder Society-sponsored revision of the Unified Parkinson's Disease Rating Scale III, MDS-UPDRS III), nonmotor symptoms (nonmotor symptoms questionnaire, NMS-Quest), and QoL (PD Questionnaire-39, PDQ-39) were analysed in 93 PD patients without dementia. Multivariable and multivariate analyses were performed to study the association between clinical parameters and PDQ-39 domains.

**Results:**

Among the autonomic symptoms, sicca symptoms (xerostomia and dry eyes) were the most commonly reported (69%), followed by sexual dysfunction in men, leaking of urine, vasomotor dysfunction, constipation, sudomotor dysfunction, and orthostatic symptoms. The autonomic symptom burden (SAS total) correlated with the NMS-Quest and Hoehn and Yahr stage, but not with age, levodopa equivalent daily dose, disease duration, and the MDS-UPDRS III. The SAS total score was an independent predictor of the PDQ-39 summary index and mainly affected the PDQ-39 cognition and emotional well-being domains. Sicca symptoms were not associated with age, MDS-UPDRS III, disease duration, Hoehn and Yahr stage, and levodopa equivalent daily dose but aggravated the PDQ-39 domains: cognition, emotional well-being, bodily discomfort, and mobility. Sicca symptoms frequently occur together with other nonmotor symptoms, namely, urine urgency, orthostatic problems, and concentration problems. Overall, 75% of the subjects took at least one drug that can cause sicca symptoms (anti-PD medication, antidepressant drugs, antihypertensive drugs, antipsychotic drugs, antimuscarinic drugs, and analgesic drugs).

**Conclusion:**

Sicca symptoms are common in PD and negatively influence QoL. The observed association between sicca symptoms and other nonmotor symptoms provides further preliminary evidence for the growing recognition of different nonmotor clusters in PD.

## 1. Introduction

Nearly every patient with Parkinson's disease (PD) suffers from nonmotor symptoms [1]. Among these nonmotor symptoms, the impairment of the autonomic nervous system (e.g., tear, salivary, cardiovascular, urinary, and sexual functions) is commonly yet underrecognised [2, 3]. In a study with 135 PD patients, 84% of the patients complained of autonomic symptoms, according to the Scales for Outcomes in Parkinson's disease-Autonomic Dysfunction (SCOPA-AUT) [4]. The severity of autonomic symptoms was associated with higher age, longer disease duration, greater disease severity, and higher doses of dopaminergic medication [4, 5]. However, in another study of 40 patients with PD, autonomic dysfunction did not correlate with age or motor symptoms [6]. Dysautonomia was also found to be associated with impaired health-related quality of life (QoL) and disease progression [7, 8]. Many aspects of QoL (e.g., activity of daily living, emotion, cognitive functions, communication, and social support) can be affected by autonomic dysfunction [6].

However, the frequently used SCOPA-AUT is heavily weighted toward gastrointestinal and urinary functioning and misses aspects such as xerostomia or sudomotor functioning. We therefore studied autonomic syndromes in PD by using the Survey of Autonomic Symptoms (SASs). By doing so, we aimed to explore the association between autonomic symptoms, sicca symptoms (xerostomia and dry eyes), PD-specific clinical parameters, and health-related QoL [9, 10].

## 2. Methods

### 2.1. Study Design and Assessments

In this observational study, patients with PD were recruited consecutively from the Department of Neurology ward, Jena University Hospital, Jena, Germany. All patients came as planned to the hospital and received multimodal treatment by specialised therapists and medication modifications during their stay (German Multimodale Komplexbehandlung bei Morbus Parkinson). The patients were admitted for the following reasons: an increase in fluctuations, worsening dyskinesias, an increase in off-phases, evaluation for deep brain stimulation, and worsening of gait and freezing. The following categories of patients were excluded: current smokers and those suffering from diabetes mellitus, Sjögren syndrome in the anamnesis, and chemotherapy, radiation, or malignant disorders. The Movement Disorder Society-sponsored revision of the Unified Parkinson's Disease Rating Scale III (MDS-UPDRS III) [11], the revised nonmotor symptoms questionnaire (NMS-Quest), and Hoehn and Yahr staging were used to evaluate motor and nonmotor symptoms. Cognition was assessed by the Montreal Cognitive Assessment (PD dementia <21 points). The PD Questionnaire-39 (PDQ-39) was used to assess health-related QoL [12]. The PDQ-39 comprises 39 questions with five different answer options (never, occasionally, sometimes, often, or always) and covers eight domains: mobility, activities of daily living (ADL), emotional well-being, stigma, social support, cognition, communication, and bodily discomfort. The PDQ-39 summary index (SI) was obtained by calculating the mean of the eight domain scores. Higher scores indicate poorer health-related QoL, with the maximum score of 100, indicating the worst level of problems. Autonomic symptoms were assessed using the German SAS consisting of 11 items in women and 12 items in men [9, 10], covering the following autonomic symptom domains: orthostatic, sudomotor, vasomotor, gastrointestinal, urinary, and sexual dysfunction. The questions require a yes (1 point) or no (0 point) response to symptoms occurring in the 6 month period prior to administration (SAS A subscore). The maximum number of symptoms reported 0–12 for men and 0–11 for women. For the total symptom impact score, each item is then rated on a Likert scale ranging from 1 (least severe) to 5 (most severe) to determine the total autonomic symptom impact score (SAS total) as the sum of all scores (0–60 for men and 0–55 for women).

### 2.2. Statistical Analyses

The SPSS statistical computer package (version 23.0; IBM Corporation, Armonk, NY, USA) was used for all statistical analyses. All continuous data are presented as mean and standard deviation (SD) or median and interquartile range. Categorical variables are presented as numbers and percentages (%). Descriptive analyses were used to describe the frequency of autonomic symptoms. Pearson's and Spearman's correlations were used to evaluate the relationship between the SAS total score and clinical parameters. We performed a multivariable analysis using a linear regression model with stepwise forward selection (Akaike information criterion) to evaluate the factors independently related to PDQ-39 SI (dependent variable) with SAS total score, MDS-UPDRS III, gender, age, NMS-Quest, and Hoehn and Yahr stage as independent variables. To study the impact of the SAS total score on the PDQ-39 domains (with and without inclusion of NMS-Quest as covariate), a multivariate analysis was used. Group comparisons between patients with and without sicca symptoms (second SAS item ≥ 0) were performed using the *t*-test, *U*-test, or *χ*^2^ test. In the multivariate analysis, the association between sicca symptoms (present or absent) and PDQ-39 domains was studied. The similarity between presence/absence of sicca symptoms and presence/absence of different nonmotor symptoms in the NMS-Quest was measured using Russell and Rao binary similarity measures; here, equal weight is given to matches and nonmatches. For all analyses, the statistical significance was set to *p* < 0.05.

## 3. Results

### 3.1. Descriptive Statistics

**Figure 1:**
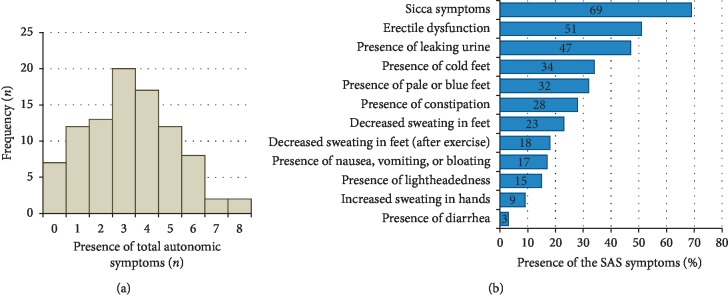
(a) The histogram shows the frequency of autonomic symptoms according to the Survey of Autonomic Symptoms (SASs) A subscore. Accordingly, the patients had a mean of 3.3 (SD 1.9) (range 0–8) autonomic symptoms. (b) Percentage of positive items in the SAS (in descending frequency order). Among the autonomic symptoms assessed with the SAS, the sicca symptoms were the most commonly reported followed by sexual dysfunction in men, leaking of urine, vasomotor dysfunction, constipation, sudomotor dysfunction, and orthostatic symptoms.

One hundred patients with PD were assessed, and after exclusion of subjects with PD dementia, the data from 93 patients were used for the following analyses. Clinical parameters are detailed in Table 1. According to the SAS questionnaire, the patients in our cohort had a mean of 3.3 (SD 1.9) (range 0–8) autonomic symptoms (Figure 1(a)). Among these, sicca symptoms (xerostomia and dry eyes) were the most commonly reported followed by sexual dysfunction in men, leaking of urine, vasomotor dysfunction, constipation, sudomotor dysfunction (items 5–7), and orthostatic symptoms (Figure 1(b)).

### 3.2. Association between Total Autonomic Disease Burden and PDQ-39

The mean total SAS was 10.5 (SD 6.7). As indicated by the correlation coefficients, the SAS total correlated moderately with the NMS-Quest (*r* = 0.48, *p* < 0.001) and weakly with the Hoehn and Yahr stage (*r* = 0.31, *p*=0.02), but not with age, LEDD, disease duration, and the MDS-UPDRS III.

In the multivariable analysis, the SAS total score was found to be an independent predictor of health-related QoL (PDQ-39 SI) (Table 2). The multivariate analysis revealed a significant association between the SAS total score and the PDQ-39 domains (*p* < 0.001; Wilk's Λ = 0.59, partial *η*^2^ = 0.41) with descending impact on cognition (partial *η*^2^ = 0.221, *p* < 0.001), emotional well-being (partial *η*^2^ = 0.163, *p* < 0.001), social support (partial *η*^2^ = 0.122, *p*=0.001), communication (partial *η*^2^ = 0.105, *p*=0.002), bodily discomfort (partial *η*^2^ = 0.103, *p*=0.003), mobility (partial *η*^2^ = 0.102, *p*=0.003), and ADL (partial *η*^2^ = 0.078, *p*=0.009) (for stigmatisation *p*=0.21). After correction for the NMS-Quest in the multivariate analysis, only the association between SAS and the PDQ-39 domain cognition remained significant (partial *η*^2^ = 0.14, *p*=0.002).

### 3.3. Association between Sicca Symptoms, Clinical Parameters, and PDQ-39

The patients with and without sicca symptoms (second SAS item >0 or =0) did not differ in terms of age, MDS-UPDRS III, disease duration, Hoehn and Yahr stage, and LEDD. However, the patients with sicca symptoms had more nonmotor symptoms (mean NMS-Quest = 12.3, SD = 4.1, 95% CI [11.2–13.5]) in comparison to the patients without sicca symptoms (mean NMS-Quest 6.2, SD = 3.2, 95% CI [4.7–7.7]). The patients with sicca symptoms were characterised by poorer health-related QoL, as indicated by higher values for the PDQ-39 domains ADL, emotional well-being, cognition, and bodily discomfort (Table 3). In the multivariate analysis, sicca symptoms were associated with higher PDQ-39 scores (worse health-related QoL) in the following domains: cognition (partial *η*^2^ = 0.14, *p*=0.001), emotional well-being (partial *η*^2^ = 0.12, *p*=0.001), bodily discomfort (partial *η*^2^ = 0.11, *p*=0.002), and mobility (partial *η*^2^ = 0.08, *p*=0.01) (*p* < 0.006; Wilk's Λ = 0.82, partial *η*^2^ = 0.18).

**Figure 2:**
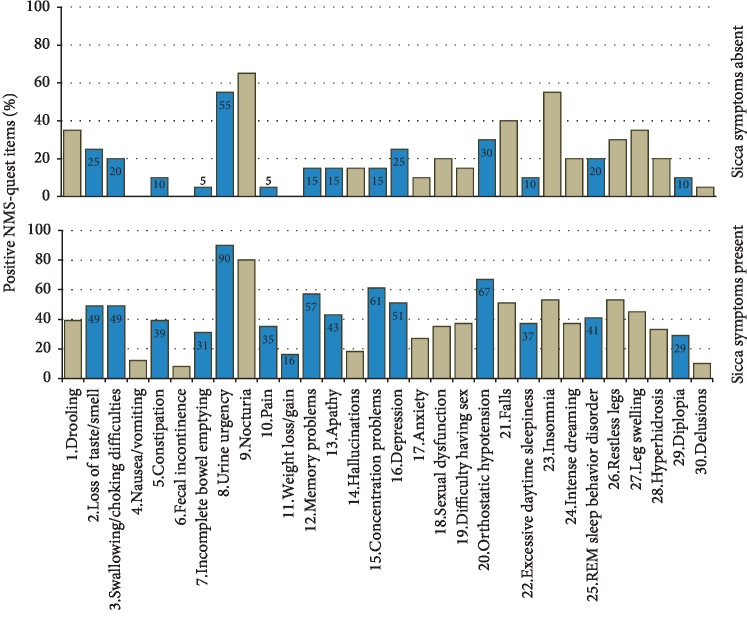
Prevalence of nonmotor symptoms in patients with and without sicca symptoms. For significant group differences (*χ*^2^ test), the values are provided in the blue columns.

**Figure 3:**
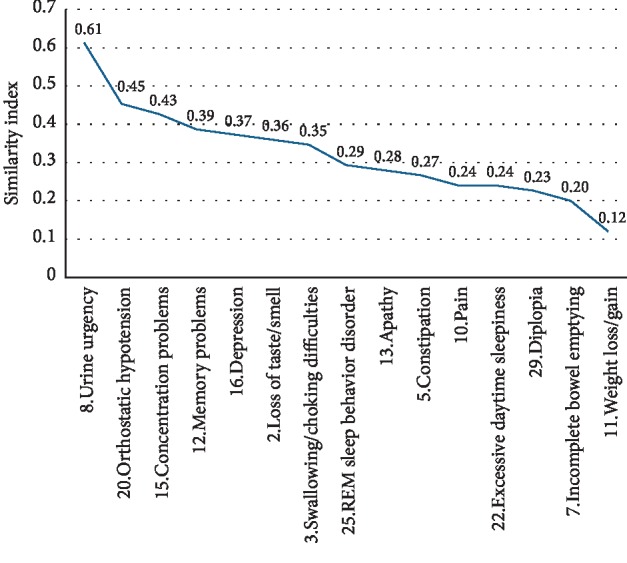
Similarity index between sicca symptoms and nonmotor symptoms questionnaire (NMS-Quest) items using the Russel–Rao binary similarity measures.

Distinct nonmotor symptoms according to the NMS-Quest were more common in patients with sicca symptoms in comparison to patients without sicca symptoms: loss of taste/smell (*χ*^2^, *p*=0.04), swallowing difficulties or choking (*p*=0.03), constipation (*p*=0.04), incomplete bowel emptying (*p*=0.008), urine urgency (*p* < 0.001), pain (*p*=0.007), weight loss/gain (*p*=0.03), memory problems (*p*=0.001), apathy (*p*=0.01), concentration problems (*p* < 0.001), depression (*p*=0.01), orthostatic hypotension (*p*=0.015), excessive daytime sleepiness (*p*=0.01), REM sleep behaviour disorder (*p*=0.04), and diplopia (*p*=0.027) (Figure 2). Finally, we explored the cooccurrence of sicca symptoms with these nonmotor symptoms. Here, the highest binary similarity with the presence of sicca symptoms was observed for urine urgency, followed by orthostatic problems and concentration problems (Figure 3).

### 3.4. Sicca Symptoms and Prescribed Medications

**Figure 4:**
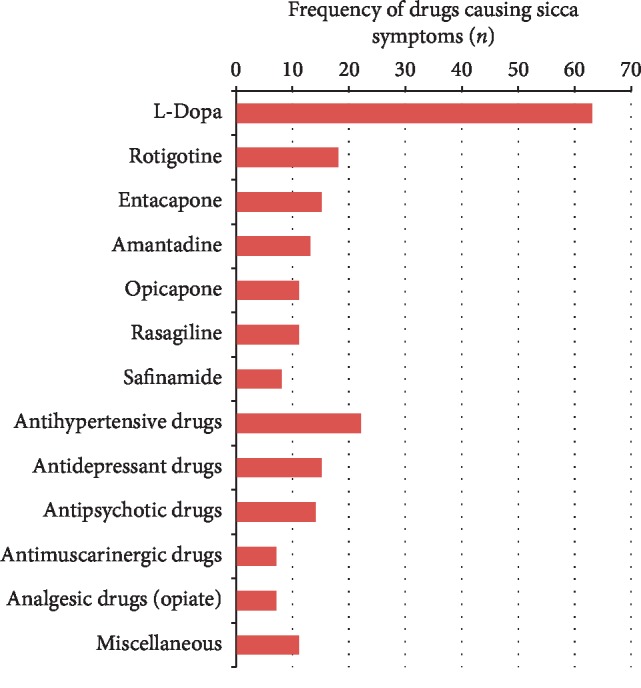
Frequency of drugs causing sicca symptoms in the cohort. The most commonly prescribed drugs, which can cause sicca symptoms, were anti-PD medications (*n* = 139) followed by antidepressant drugs (mirtazapine, venlafaxine, and citalopram), antihypertensive drugs (bisoprolol, amlodipine, and hydrochlorothiazide), antipsychotic drugs (quetiapine and clozapine), antimuscarinic drugs for overactive bladder (solifenacin and darifenacin and trospium chloride), analgesic drugs (morphine, tramadol, and tilidine) and other miscellaneous drugs (gabapentin, pregabalin, and pantoprazole).

Finally, the drugs prescribed to the patients were analysed, and those that could cause sicca symptoms as side effects were counted. On average, each patient took 2.6 drugs that could cause sicca symptoms (SD = 1.3, range 0–6). Only 12 patients took no drugs that could cause sicca symptoms. Most patients took one (*n* = 13), two (*n* = 28), three (*n* = 19), four (*n* = 17), five (*n* = 3), or six (*n* = 1) drugs that could cause sicca symptoms. As expected, the anti-PD drugs accounted for the largest share, followed by antidepressant drugs (8 × mirtazapine, 7 × venlafaxine, 7 × citalopram, and 1 × opipramol), antihypertensive drugs (15 × bisoprolol, 4 × amlodipine, and 3 × hydrochlorothiazide), antipsychotic drugs (10 × quetiapine and 4 × clozapine), antimuscarinic drugs for the overactive bladder (3 × solifenacin, 1 × darifenacin, and 3 × trospium chloride), analgesic drugs (4 × morphine, 1 × tramadol, and 2 × tilidine), and other miscellaneous drugs (6 × gabapentin, 2 × pregabalin, and 3 × pantoprazole) (Figure 4).

## 4. Discussion

In the present study, we explored the relationships between autonomic symptoms, other nonmotor symptoms, and health-related QoL in 93 PD patients without dementia.

Most of the patients reported at least one autonomic symptom. In line with former studies, we observed a high frequency of sexual and urinary dysfunction [13–15]. In accordance with other studies, age and disease duration did not influence the severity of autonomic dysfunction [6, 16]. As expected, we observed more autonomic symptoms in patients with higher disease stages and higher NMS-Quest. Corresponding to the literature, autonomic syndromes worsen health-related QoL, as assessed by the PDQ-39 SI [3, 6, 17, 18]. Our study confirms and details this observation. The total autonomic symptom burden was associated with poorer health-related QoL in several domains of the PDQ-39. In particular, cognition was negatively influenced by autonomic symptoms. To interpret this result, it is noteworthy that the cognition domain shows a stronger relationship to mood, especially depression, than to measure of cognitive function [19]. The cognitive domain shows some overlap with the emotional well-being domain, which is primarily influenced by symptoms of depression and long-standing anxiety. Thus, mood disorders are related to autonomic symptoms. Because our study was not intended to explore the causal relationship, we cannot answer whether this is a primary relationship among PD, mood disorders, and autonomic disturbances or a secondary effect, e.g., due to the side effects of medication. However, independent of the aetiology, it is important for the clinician to be aware that the presence of autonomic symptoms is related to poorer health-related QoL via mood disturbances.

Although the majority of previous studies found gastrointestinal and urinary dysfunctions to be the most common autonomic syndromes [13, 14], in our cohort, sicca symptoms (xerostomia and dry eyes) were the most commonly found autonomic syndromes. This is mainly because the frequently used SCOPA-AUT does not cover sicca symptoms. Moreover, many elderly patients, as well as PD patients, receive polymedication with anticholinergic side effects, which is an avoidable reason for xerostomia [20, 21]. This was also true for our study where 75% of the subjects took at least one drug that can cause sicca symptoms. The commonly prescribed medications in our study that can cause sicca symptoms were anti-PD medication (mainly levodopa), antidepressant drugs, antihypertensive drugs, antipsychotic drugs, antimuscarinic drugs, and analgesic drugs.

Xerostomia is an abnormal subjective feeling of dry mouth. Because it can favour both caries and periodontal disease, it is highly relevant for PD sufferers who are at risk of developing malnutrition [22, 23]. In general, 30% of elderly people complain about dry mouth with significant and negative impacts on QoL [24, 25]. In the study by Barbe et al., 50% of the PD patients reported xerostomia [26]. Here, and in another study, the German Oral Health Impact Profile-14 indicated worse oral health-related QoL in PD patients with xerostomia [27]. Our study extends these findings and shows that sicca symptoms do not only influence oral health but also general health-related QoL. Moreover, our results show that sicca symptoms mainly influence the domains of cognition and emotional well-being and are therefore also associated with mood disturbances in PD. Further studies are necessary to explore the causal relationship between sicca symptoms and mood disturbances in longitudinal studies. Given the high impact on health-related QoL, it is nevertheless important to actively ask patients about these symptoms and provide specific therapy [28].

With the growing recognition of NMS-dominant presentation of PD [29], it is of interest that we observed some associations between sicca symptoms and other nonmotor symptoms [30]. In our study, sicca symptoms were commonly accompanied by urine urgency, orthostatic problems, and concentration problems. A recent study identified six nonmotor clusters in PD [30]. Urine urgency, concentration, and memory problems belonged to one cluster and orthostatic problems to another cluster in this study by Mu et al. On the contrary, concentration problems were found to be associated with orthostasis [31, 32]. Because sicca symptoms were not included in the cluster analysis by Mu et al. and because our study did not perform cluster analysis of nonmotor symptoms and autonomic symptoms, further studies are necessary to explore the significance of sicca symptoms in terms of nonmotor clusters.

The study is not free of limitations. This monocentric study was restricted to hospitalised patients without dementia, limiting the generalisability of the results. Because we were interested in the influence of autonomic symptoms in general and especially the impact of sicca symptoms on QoL, the study was not designed to make causal statements. However, we believe that the observed associations between sicca symptoms and other nonmotor symptoms can serve as a basis for further studies exploring nonmotor subtypes in PD.

## 5. Conclusion

Although sicca symptoms are common in PD, they are still underreported in the literature. Sicca symptoms are associated with poor health-related QoL mainly via the worsening of cognitive and emotional well-being domains. In particular, the relevance of sicca symptoms in terms of nonmotor subtypes and progression needs further studies.

## Figures and Tables

**Table 1:** Demographic and clinical characteristics (*n* = 93).

Age (mean, SD; years)	66.9	10.1
Disease duration (mean, SD; months)	116.13	81.0
Sex (no., %)		
Female	34	36.6
Male	59	63.4
Hoehn and Yahr (median, IQR)	3.0	2
Survey of autonomic symptoms subscore A(mean, SD)	3.3	1.9
Survey of autonomic symptoms total score (mean, SD)	10.5	6.7
MDS-UPDRS III (mean, SD)	31.4	13.9
NMS-Quest (mean, SD)	10.6	4.7
Levodopa equivalent daily dose (mean, SD, mg)	773	695
Health-related QoL (mean, SD)		
(i) PDQ-39 mobility	50.9	29.0
(ii) PDQ-39 activities of daily living	39.2	26.6
(iii) PDQ-39 emotional well-being	34.5	24.5
(iv) PDQ-39 stigmatisation	19.0	23.3
(v) PDQ-39 social support	15.5	18.4
(vi) PDQ-39 cognition	34.4	21.7
(vii) PDQ-39 communication	26.1	21.9
(viii) PDQ-39 bodily discomfort	36.7	25.9
(ix) PDQ-39 summary index	31.9	16.9

*Abbreviations.* MDS-UPDRS, Movement Disorder Society-sponsored revision of the unified Parkinson's Disease Rating Scale; NMS-Quest, revised nonmotor symptoms questionnaire; PDQ-39, Parkinson's disease quality of life questionnaire.

**Table 2:** Multivariable analysis of predictors of health-related quality of life (PDQ-39 summary index).

Overall model	Adjusted *R*^2^ = 0.42 (*p* < 0.001)	
Independent variables	*Β*	*p*
(i) NMS-Quest	0.57	<0.001
(ii) SAS total	0.19	0.006
(iii) Gender	0.14	0.016
(iv) MDS-UPDRS III	0.10	0.042

**Table 3:** Group comparison of subjects with and without sicca symptoms.

	Subjects without sicca symptoms		Subjects with sicca symptoms		*p*
Sex (no., %)					
Female	6		28		0.03
Male	23		36		
Age (mean, SD; years)	65.7	9.1	67.5	10.5	0.44
Survey of autonomic symptoms total score (mean, SD)	5.4	4.9	12.8	6.1	<0.001
Hoehn and Yahr (median, IQR)	2.5	1	3	1	0.11
MDS-UPDRS III (mean, SD)	31.1	12.9	31.5	14.3	0.89
NMS-Quest (mean, SD)	6.2	3.1	12.3	4.1	<0.001
Disease duration (mean, SD; months)	94.5	74.5	127.1	82.6	0.09
Levodopa equivalent daily dose (mean, SD, mg)	567	431	874	778	0.11
Health-related QoL (mean, SD)					
(i) PDQ-39 mobility	38.1	32.7	56.5	25.6	0.007
(ii) PDQ-39 activities of daily living	28.9	25.3	43.6	26.1	0.015
(iii) PDQ-39 emotional well-being	20.9	22.8	40.4	23.0	<0.001
(iv) PDQ-39 stigmatisation	12.1	18.2	22.0	24.8	0.06
(v) PDQ-39 social support	10.7	16.1	17.7	19.1	0.10
(vi) PDQ-39 cognition	22.0	17.2	39.7	21.3	<0.001
(vii) PDQ-39 communication	21.8	24.6	28.0	20.6	0.22
(viii) PDQ-39 bodily discomfort	23.7	23.5	42.1	25.1	0.002
(ix) PDQ-39 summary index	22.3	16.1	35.9	15.8	0.001

*Abbreviations.* MDS-UPDRS, Movement Disorder Society-sponsored revision of the Unified Parkinson's Disease Rating Scale; NMS-Quest, revised nonmotor symptoms questionnaire; PDQ-39, Parkinson's disease quality of life questionnaire.

## Data Availability

The data used to support the findings of this study are available from the corresponding author upon request for scientific purposes only.
